# The role of DNA methylation in ovarian cancer chemoresistance: A narrative review

**DOI:** 10.1002/hsr2.1235

**Published:** 2023-04-27

**Authors:** Kaiyang Song, Mara Artibani

**Affiliations:** ^1^ Green Templeton College University of Oxford Oxford UK; ^2^ Ovarian Cancer Cell Laboratory, Medical Research Council (MRC) Weatherall Institute of Molecular Medicine University of Oxford Oxford UK; ^3^ Nuffield Department of Women's & Reproductive Health University of Oxford Oxford UK

**Keywords:** chemoresistance, epigenetics, methylation, ovarian cancer

## Abstract

**Background and Aims:**

Ovarian cancer (OC) is the most lethal gynecological cancer. In 2018, it was responsible for over 180,000 deaths worldwide. The high mortality rate is the culmination of a lack of early diagnosis and high rates of chemotherapy resistance, which is synonymous with disease recurrence. Over the last two decades, an increasingly significant role of epigenetic mechanisms, in particular DNA methylation, has emerged. This review will discuss several of the most significant genes whose hypo/hypermethylation profiles are associated with chemoresistance. Aside from functionally elucidating and evaluating these epimutations, this review will discuss recent trials of DNA methyltransferase inhibitors (DNMTi). Finally, we will propose future directions that could enhance the feasibility of utilizing these candidate epimutations as clinical biomarkers.

**Methods:**

To perform this review, a comprehensive literature search based on our keywords was conducted across the online databases PubMed and Google Scholar for identifying relevant studies published up until August 2022.

**Results:**

Epimutations affecting MLH1, MSH2, and Ras‐association domain family 1 isoform A (DNA damage repair and apoptosis); ATP‐binding cassette subfamily B member 1 and methylation‐controlled J (drug export); secreted frizzled‐related proteins (Wnt/β‐catenin signaling), neurocalcin delta (calcium and G protein‐coupled receptor signaling), and zinc finger protein 671 all have potential as biomarkers for chemoresistance. However, specific uncertainties relating to these epimutations include histotype‐specific differences, intrinsic versus acquired chemoresistance, and the interplay with complete surgical debulking. DNMTi for chemoresistant OC patients has shown some promise; however, issues surrounding their efficacy and dose‐limiting toxicities remain; a personalized approach is required to maximize their effectiveness.

**Conclusion:**

Establishing a panel of aberrantly methylated chemoresistance‐related genes to predict chemoresponsiveness and patients' suitability to DNMTi could significantly reduce OC recurrence, while improving DNMTi therapy viability. To achieve this, a large‐scale prospective genome‐wide DNA methylation profile study that spans different histotypes, includes paired samples (before and after chemotherapy), and integrates transcriptomic and methylomic analysis, is warranted.

## 1 INTRODUCTION

Over the last three decades, there has been an average increase in 5‐year survival rates of 20% across all cancers.^1^ However, such improvements have not been observed in ovarian cancer (OC): this “silent killer” remains the most lethal gynecological cancer with a 5‐year survival rate of 45%.^2^ Epithelial ovarian cancer (EOC) is the most common type, constituting 85% of all cases and comprising four main histotypes: serous, endometrioid, clear‐cell, and mucinous.^3^ EOC has traditionally been treated through surgical tumor debulking followed by combined platinum/taxane therapy—primarily carboplatin and paclitaxel. In cases of advanced, platinum‐sensitive OC, recent NICE guidance has approved the use of Olaparib (PARP inhibitor) alongside bevacizumab for treating tumors that exhibit homologous recombination deficiency.^4^ The utility of Olaparib as a maintenance therapy has been widely documented across current literature,^5^ but is beyond the scope of this review.

Platinum therapies induce interstrand and intrastrand DNA adducts, which trigger mismatch repair (MMR) pathways and apoptosis.^6^ Taxanes bind β‐tubulin to prevent microtubule depolymerization, in turn, promoting cell cycle arrest and apoptosis.^7^ Although 70% of OC patients initially respond to chemotherapy, subsequent chemoresistance and disease recurrence remain significant concerns and underpin 90% of eventual deaths.^8^

For several decades, it has been well‐documented that genetic mutations affecting a plethora of processes including DNA repair, epithelial–mesenchymal transition (EMT), and drug export, are implicated in chemoresistance.^9^ Nevertheless, in some instances, it appears that genetic alterations alone may not fully elucidate chemoresistance.^10^, ^11^ Thus, an important role of epigenetic mechanisms has emerged. Since an initial association between DNA methylation, the most common epigenetic alteration, and human cancer was established in 1983,^12^ the development of novel technologies based on methylation arrays (e.g., Illumina's HumanMethylation450k) and methylation sequencing (e.g., reduced representation bisulfite sequencing),^13^ have facilitated a greater understanding of aberrant DNA methylation in OC chemoresistance.

DNA methylation is mediated by DNA methyltransferases (DNMT1, DNMT3a, DNMT3b) which add methyl groups to cytosine residues of CpG dinucleotides. Tumor cells generally exhibit global DNA hypomethylation (particularly across repetitive DNA sequences), but de novo hypermethylation at CpG islands. This latter phenomenon often occurs at promoter sites of cancer‐related genes, typically downregulating their expression.^14^ Studies have shown that hypermethylation at CpG islands of tumor suppressor genes is associated with earlier disease recurrence and reduced progression‐free survival in OC patients.^15^, ^16^ Inspired by such findings, over the last two decades, various studies have highlighted a range of hypo/hypermethylated candidate genes that are associated with OC chemoresistance (Table 1).

**Table 1:** Selected genes whose aberrant promoter methylation statuses are associated with chemoresistant ovarian cancer.

Gene	Hypo‐/hypermethylation	Change in gene expression	Biological process	Resistance against	References		
ABCB1	Hypomethylation	Upregulation	Drug export	Multidrug resistance	[17, 18]		
ABCG2	Hypomethylation	Upregulation	Drug export	Multidrug resistance	[19]		
BRCA1	Hypermethylation	Downregulation	DNA damage response	Platinum	[20, 21]		
CDH2	Hypermethylation	Downregulation	Cell adhesion	Platinum	[22]		
c‐FOS	Hypermethylation	Downregulation	c‐Fos/AP‐1 signaling	Platinum	[23]		
CLDN4	Hypomethylation	Upregulation	Cell invasion, beta‐catenin signaling	Platinum	[24, 25]		
DACT1	Hypermethylation	Downregulation	Wnt signaling	Platinum	[26]		
ITFAV	Hypermethylation	Downregulation	Cell adhesion	Platinum	[22]		
MCJ	Hypermethylation	Downregulation	ABCB1 inhibition	Multidrug resistance	[27, 28]		
MEST	Hypermethylation	Downregulation	Wnt signaling	Platinum	[29]		
MLH1	Hypermethylation	Downregulation	DNA mismatch repair, apoptosis	Platinum	[30, 31, 32, 33]		
MSH2	Hypermethylation	Downregulation	DNA mismatch repair, apoptosis	Platinum	[34, 35]		
NAGA	Hypermethylation	Downregulation	Apoptosis	Platinum	[36]		
NEO1	Hypermethylation	Downregulation	Cell adhesion	Platinum	[22]		
PTEN	Hypermethylation	Downregulation	Apoptosis, PI3K/AKT signaling^37^	Taxane	[38]		
RASSF1A	Hypermethylation	Downregulation	Ras signaling, apoptosis, microtubule stabilization	Taxane	[20, 39, 40]		
RGS10	Hypermethylation	Downregulation	Apoptosis	Platinum	[41]		
SERPINE1	Hypomethylation	Upregulation	Cell adhesion, invasion, regulates levels of vimentin, snail, and twist	Platinum	[42]		
SFRPs (SFRP1‐5)	Hypermethylation	Downregulation	Wnt signaling, cell proliferation, and invasion	Platinum	[43, 44, 45, 46]		
SLC6A14	Hypomethylation	Upregulation	Drug uptake/efflux	Platinum	[47]		
TMEM88	Hypomethylation	Upregulation	Wnt signaling	Platinum	[48]		
TRIB2	Hypermethylation	Downregulation	MAPK‐ and Wnt‐ signaling	Platinum	[23]		
VIM	Hypomethylation	Upregulation	Cell migration, attachment, and invasion	Platinum	[49]		

Although methylation can occur at intergenic regions, this review will focus on methylation events occurring at the promoter sites of EOC resistance‐associated genes. We will discuss and evaluate genes that are involved in well‐established resistance‐related processes/pathways, including drug export (ATP‐binding cassette subfamily B member 1 [ABCB1], methylation‐controlled J [MCJ]), DNA repair and apoptosis (MLH1, MSH2, Ras‐association domain family 1 isoform A [RASSF1A]), Wnt/β‐Catenin signaling (secreted frizzled‐related proteins [SFRPs], Zinc finger protein 671 [ZNF671]), and calcium signaling (neurocalcin delta [NCALD]). With each biomarker, we will discuss how specific hypo/hypermethylation events manifest at the cellular level vis‐à‐vis changes in gene expression and tumor cell phenotype, as well as the clinical impact of these epimutations. Finally, given the current lack of accurate clinical biomarkers to predict OC chemosensitivity, this review will propose important experiments which could help identify a panel of methylation profiles that stratifies OC patients based on predicted chemosensitivity. Indeed, these investigations could simultaneously enhance the clinical viability of DNMTi.

### 1.1 Methodology

A comprehensive search, based on our keywords, was conducted on the PubMed database and Google Scholar from February 2021 to August 2022. Our selected terms included “Ovarian cancer,” “chemoresistance,” “methylation,” “epigenetics,” and “DNA methyltransferase inhibitor.” The literature search strategy for the data in tables and results is provided in Supporting Information: Figure S1.

The set inclusion criteria for selected articles were (a) original studies (including preclinical studies), observational and interventional studies, or randomized controlled trials; (b) articles with full‐text accessibility; (c) articles in the English language. We excluded articles that were (a) case reports, editorials, reviews, and commentaries; (b) describing nonmethylation epimutations or subtypes of OC other than EOC; (c) abstract‐access only; (d) published in languages other than English.

## 2 MAIN TEXT

### 2.1 Drug export

#### 2.1.1 ABCB1

**Figure 1:**
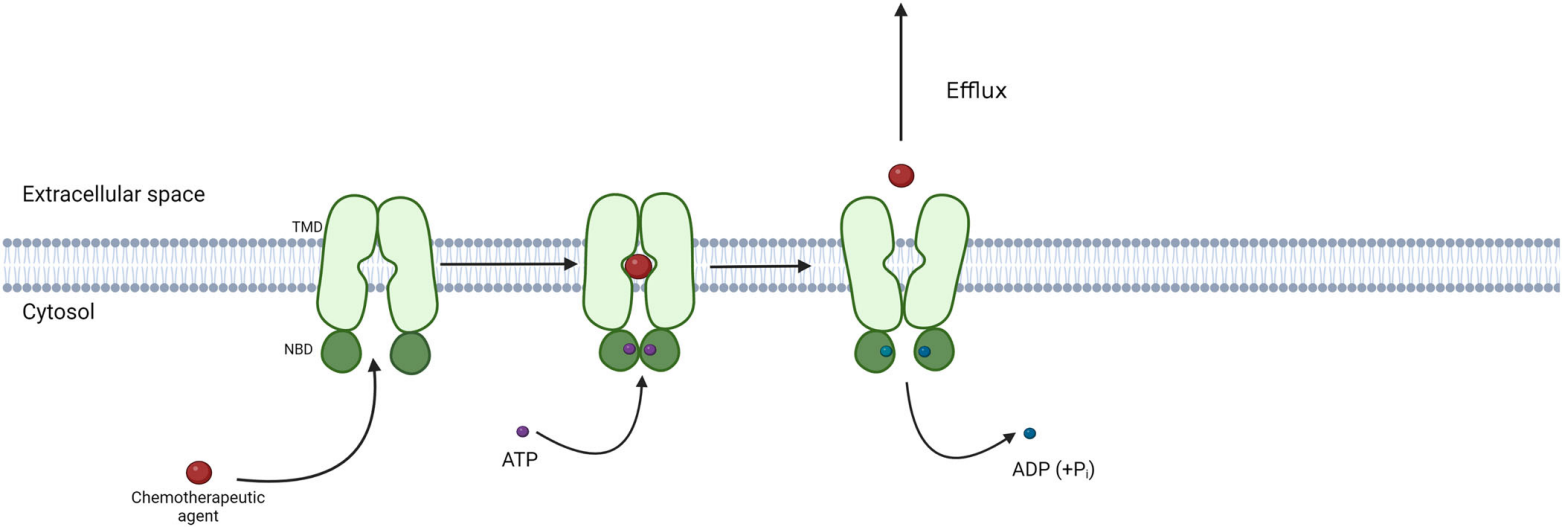
Schematic showing the mechanism of action of ATP transporters, in this case an exporter. ATP transporters typically comprise two nucleotide‐binding domains (NTD) and two transmembrane domains (TMD). After the chemotherapeutic drug binds the TMDs, the energy released from ATP hydrolysis facilitates a conformational change to an outward‐facing conformation and, in turn, drug exportation. [Created with BioRender.com].

ABC transporters represent a critical facet of chemoresistance as they can prevent the accumulation of chemotherapeutic drugs in tumor cells (Figure 1). Oligonucleotide microarray analysis has revealed that ABCB1 and ABCG2 are prominently upregulated in chemoresistant W1 OC cell lines.^50^ Subsequently, DAVID analysis of aberrantly methylated genes in chemoresistant OC tumors showed enrichment of ABCB1 and ABCG2 in drug response processes.^39^ In vitro experiments have united these findings and shown that hypomethylation and subsequent upregulated expression of ABCB1 and ABCG2 are associated with chemoresistance.^17^, ^19^ ABCB1 is the most well‐investigated ABC transporter in OC; notably, in chemoresistant A2780 OC cells, verapamil (ABCB1 inhibitor) sensitized cells to paclitaxel.^18^ Subsequent experiments involving Calcein‐AM have validated the function of ABCB1 in paclitaxel efflux. Calcein‐AM is exported via ABCB1, but hydrolysis of nonexported, intracellular Calcein‐AM generates fluorescent Calcein. Verapamil‐mediated ABCB1 inhibition increased Calcein fluorescence in a dose‐dependent manner, unlike in A2780 cells which did not express ABCB1.^18^ ABCB1 also maintains “side population” (SP) cells that possess cancer stem cell‐like characteristics including marked chemoresistant and proliferative potential. Morpholino antisense oligonucleotide‐induced silencing of ABCB1 in HeyA8MDR OC cells significantly reduced the proportion of SP cells and heightened paclitaxel sensitivity.^51^ Collectively, these in vitro findings highlight the therapeutic potential of targeting aberrant methylation of ABCB1 to curtail chemoresistance.

In accordance with these mechanistic insights, substantial evidence links ABCB1 overexpression with clinical chemoresistance and reduced time‐to‐disease progression.^52^, ^53^ Nevertheless, a relationship between ABCB1 methylation and gene expression is less well‐established in patients. Interestingly, a recent study showed ABCB1 hypermethylation in tumor samples from postchemotherapy OC patients compared to healthy patients.^54^ However, hypermethylation of ABCB1 did not correlate with reduced mRNA expression, conflicting the dogma that promoter hypermethylation downregulates gene expression. This discrepancy can be elucidated by the fact that the ABCB1 gene has an upstream and downstream promoter. Indeed, in drug‐resistant breast cancer and lung adenocarcinoma cells, downstream promoter hypermethylation enhances ABCB1 expression.^55^, ^56^ Further studies are required to investigate the methylation profile of ABCB1 in chemoresistant OC patients. Critically, these studies should integrate methylome and transcriptome analysis and should delineate between the upstream and downstream ABCB1 promoter.

However, further investigations into ABCB1 alone are insufficient: redundancy across different ABC transporters may limit the efficacy of targeting individual ABC transporters. Indeed, in human cells, paclitaxel alone can be transported by ABCC, ABCB1, ABCB4, ABCB11, ABCC1, and ABCG2.^57^ Thus, further functional and methylome characterization of these transporters and their genes would highlight the different ABC transporter‐associated resistance mechanisms employed by OC cells. Such findings would underpin the reversal of drug export gene epimutations in OC and could facilitate synergistic improvements in chemosensitivity.

#### 2.1.2 MCJ gene

Given the current ambiguity regarding ABC transporter methylation profiles in chemoresistant ovarian tumors, it may be more viable to consider the epigenetic status of ABC transporter regulators. Most notably, MCJ (part of the DnaJ cochaperone family) inhibits ABCB1 expression. MCJ was initially identified in 2001: transfecting MCJ into OC cells sensitized them to paclitaxel and cisplatin.^27^ Subsequent coimmunoprecipitation analysis revealed that MCJ binds C‐Jun (component of AP‐1 transcription factor), directing it for proteasomal degradation and preventing C‐Jun‐mediated ABCB1 expression.^58^ To that end, bisulfite sequencing revealed that hypermethylation‐mediated downregulation of MCJ expression is associated with chemoresistance both in vitro^17^ and in stage III/IV EOC patients: 17% of tumors exhibited high levels of MCJ methylation (present in >90% of clones).^28^ However, a caveat is that the role of MCJ expression in chemosensitivity has primarily been explored in serous OC. Subsequent analysis has shown differential MCJ methylation profiles across serous, clear‐cell, and endometrial OC tumors.^59^ Thus, future studies should investigate whether histology‐dependent variations in MCJ methylation impact ABCB1 expression and chemoresistance to different extents.

### 2.2 DNA repair and apoptosis

#### 2.2.1 MLH1 and MSH2

**Figure 2:**
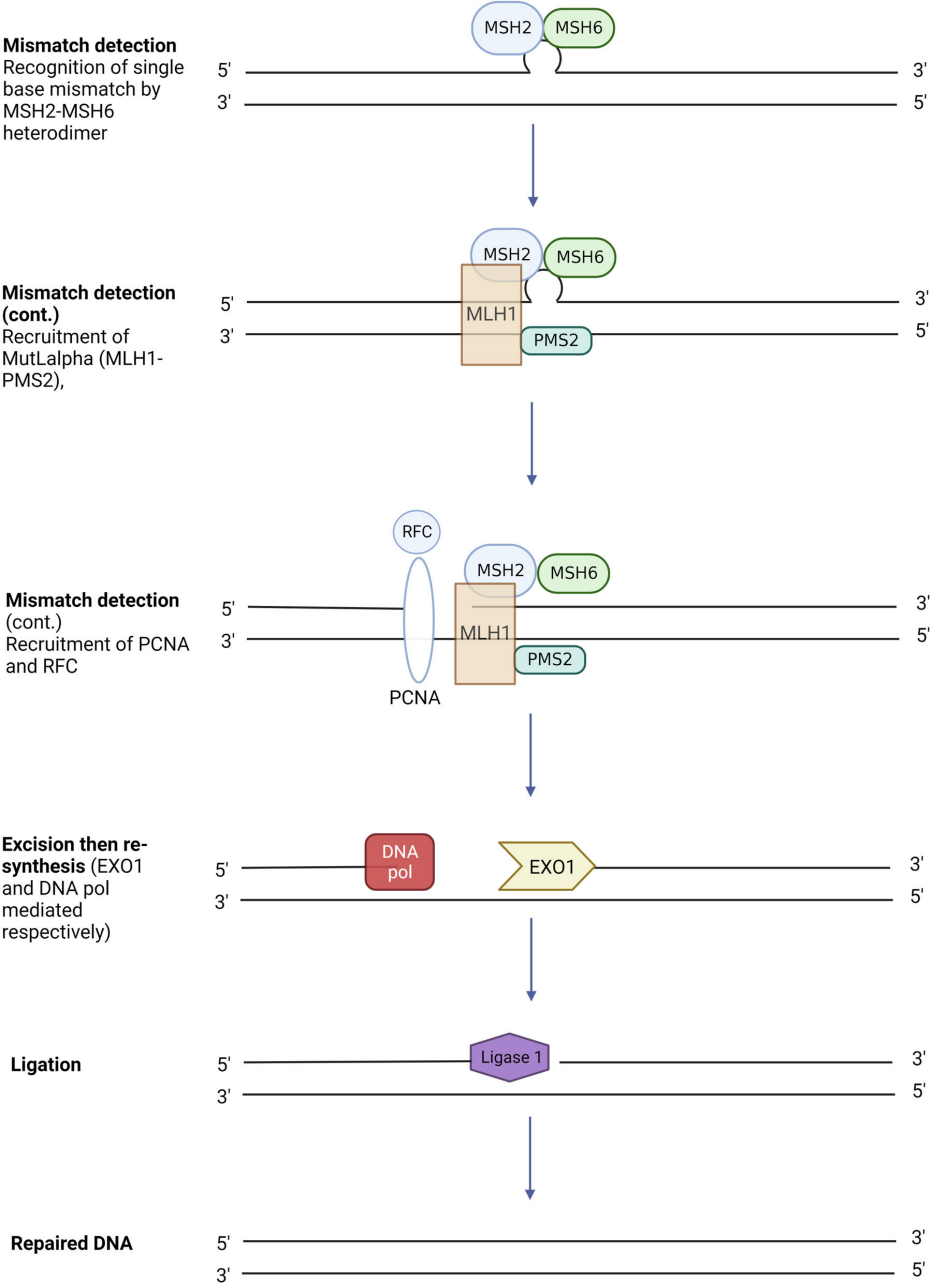
Schematic showing single base mismatch repair in human cells. In human cells MutSα (MSH2–MSH6 heterodimer) detects single base mismatches and insertion–deletion mutations. Conversely, MutSβ (MSH3/MSH6) detects longer insertion/deletion mutations. Following initial detection, the recruitment of MutLα heterodimer (MLH1–PMS2), proliferating cellular nuclear antigen (PCNA), and replication factor C (RFC) help to initiate DNA excision and resynthesis. These processes are driven by exonuclease 1 (EXO1) and DNA polymerase (DNA pol), respectively. DNA Ligase 1 seals the newly synthesized strand in term completing the DNA mismatch process. [Created with BioRender.com].

The DNA MMR proteins are critical for recognizing and repairing mismatched DNA bases (Figure 2), as well as correcting DNA polymerase slippage‐induced insertion‐deletion loops. MLH1 and MSH2, which serve in the initiation step of MMR, are the most significant MMR proteins, and their functions are frequently lost through epigenetic alterations.^60^, ^61^ Studies on cisplatin‐resistant OC cells have indicated that the downregulation of MLH1 and MSH2 can be mediated by promoter hypermethylation.^30^, ^34^ These epimutations, present in up to half of OC patients, are synonymous with defective MMR and have been associated with lymphatic metastasis, reduced overall survival, and chemoresistance.^34^, ^61^ These findings are consistent with the important roles of MLH1 and MSH2 in recognizing cisplatin‐induced DNA‐adducts and mediating apoptosis upon irreparable DNA damage.^62^ Importantly, decitabine (DNA methyltransferase inhibitors [DNMTi]) mediates MLH1 hypomethylation and subsequently resensitises chemoresistant OC xenograft models to platinum therapies.^31^ These results were replicated and elaborated in colon tumor xenografts: tumors lacking MLH1 expression (through a genetic mutation) were not sensitized by decitabine, indicating that MLH1 hypermethylation can be a “driver‐like” event during chemoresistance development.^31^ Future experiments involving DNMTi, both with and without candidate gene knockout in tumor cells/xenografts, should verify this finding in OC models and identify other hypermethylation events with “driver‐like” capacity.

Although evidence linking MLH1 and MSH2 hypermethylation with chemoresistance is robust, one study which performed methylation‐specific polymerase chain reaction (MSP) analysis on 35 OC samples did not uncover any correlation between MLH1 hypermethylation and chemosensitivity.^63^ This unexpected finding is elucidated by the fact that the study only considered patients before chemotherapy. Indeed, the prevalence of MLH1 hypermethylation among prechemotherapy OC patients is notably low,^32^, ^63^ curtailing the statistical power of such studies. To that end, investigations involving paired tumor/plasma DNA samples, before and after chemotherapy, have suggested that MLH1 hypermethylation is primarily associated with acquired chemoresistance. One study noted no initial MLH1 methylation across 36 primary OC samples, but following chemotherapy, 56.3% of tumors showed MLH1 methylation.^32^ Elsewhere, acquired MLH1 methylation following carboplatin and paclitaxel/docetaxel treatment was associated with disease recurrence and was newly identified in 27% of OC tumor DNA samples.^33^ Further paired plasma DNA studies are warranted to identify more chemosensitivity‐related genes implicated in acquired resistance, such epimutations could be monitored both during and after chemotherapy treatment.

#### 2.2.2 RASSF1A

RASSF1A is a tumor suppressor protein that is downregulated via promoter hypermethylation across various cancers, as initially observed in primary lung tumors.^64^ Early studies based on UCI‐107 Taxol‐sensitive cells showed that shRNA‐mediated RASSF1A silencing downregulates microtubule polymerization,^65^ thus facilitating resistance to paclitaxel which operates by stabilizing microtubules. Subsequently, in vitro experiments utilizing adenoviruses‐expressing RASSF1A have reinforced its important tumor suppressor function: RASSF1A upregulation reduces CyclinD1 and survivin expression,^66^ consistent with reports that RASSF1A mediates cell cycle arrest and apoptosis.^67^ Moreover, bioinformatics analysis of protein–protein interaction networks highlighted that RASSF1A interacts with the serine/threonine kinase STK4, a regulator of apoptosis.^66^, ^68^

Analysis of patient samples has revealed RASSF1A methylation is more prominent in OC tissue than in benign tissue, with marked prevalence in chemoresistant OC samples.^69^, ^70^ Nevertheless, the extent of RASSF1A promoter hypermethylation across OC patients is less well‐defined, the prevalence of this epimutation across OC cohorts has varied from 30% to almost 60%.^71^, ^72^ This variation can be elucidated by disparities in tumor histotype and patient characteristics, but also the predominant usage of MSP across studies. MSP offers a qualitative, binary assessment of methylation status and has limited accuracy when there are intermediate levels of methylation or minor methylation disparities between samples. To that end, further studies which incorporate quantitative approaches such as pyrosequencing methylation analysis, are warranted. These quantitative approaches can provide greater sensitivity and could offer a more accurate account of the methylation status of RASSF1A and other chemoresistance‐related genes.

### 2.3 Wnt/β‐catenin signaling

#### 2.3.1 SFRP family

The Wnt signaling pathway is heavily implicated in OC tumorigenesis and acts via a canonical pathway (Wnt/β‐catenin pathway) or a noncanonical pathway (β‐catenin independent). The canonical pathway is the most well‐established and has a renowned role in EMT through upregulating mesenchymal markers (e.g., Twist, Slug, and Snail), which in turn promote metastasis.^73^ Wnt/β‐catenin signaling also mediates OC chemoresistance by upregulating cellular ABCG2 expression^74^ and maintaining a population of chemoresistant stem‐like cells, known as cancer‐initiating cells.^75^ The aberrant methylation of various Wnt signaling mediators has been implicated in chemoresistance (Table 1). However, there is a lack of concordance between studies, perhaps reflecting the diversity and complexity of Wnt signaling. Nevertheless, a notable exception is the SFRP family (SFRP1–5): Wnt antagonists whose promoters are frequently hypermethylated.

While early in vitro studies showed that promoter hypermethylation of SFRPs downregulates their expression in OC,^43^, ^44^ subsequent studies have functionally characterized the role of SFRPs in tumorigenesis and chemoresistance. Overexpression of SFRP4 in A2780 cells increases cisplatin sensitivity,^76^ while EOC transgenic mouse models overexpressing SFRP1 exhibit >50% reduced tumor volume compared to WT mice.^45^ These changes were accompanied by marked β‐catenin downregulation and in the latter study, this manifested in reduced CyclinD1 and c‐Myc expression (downstream target genes implicated in cell cycle regulation). Furthermore, in chemoresistant CP70 cells, shRNA‐mediated silencing of SFRP5 enhanced cellular proliferation and invasiveness,^43^ suggestive of enhanced EMT. These results are elucidated by upregulated β‐catenin expression and Twist/Akt2 signaling: Twist/Akt2 signaling mediates paclitaxel resistance through enhancing mesenchymal markers and maintaining tumor cells with cancer stem cell‐like properties.^77^

Studies based on OC patients strongly validate these aforementioned findings. SFRP1 and SFRP2 hypermethylation have been identified in 34.9% and 62.7% of malignant OCs, respectively, and their methylation statuses are independently associated with disease recurrence.^46^ In a recent study, integrated MSP and immunohistochemical analysis showed SFRP1 promoter hypermethylation and subsequent downregulation in EOC tumor samples.^45^ A pertinent consideration here is that fallopian tube epithelium was sampled as opposed to the primary ovarian tumor. On the one hand, this approach is validated by recent work, which has shown that high‐grade serous ovarian carcinomas (HGSOC) are epigenetically similar to the precursor serous tubal intraepithelial carcinomas (STIC).^78^ Nevertheless, in up to 40% of sporadic HGSOCs there is no identifiable STIC, highlighting that fallopian tube sampling may not be a universally suitable approach.^79^ Indeed, some minor ambiguity remains over whether STICs develop into the malignant and metastatic forms of HGSOC, and also the possibility that precursor lesions originate from locations other than the fallopian tubes.^80^

Intriguingly, another study showed that although SFRP1 is downregulated in both high‐grade (HGSOC) and low‐grade serous OC (LGSOC),^81^ SFRP1 promoter hypermethylation is only prominent in HGSOC but not LGSOC. This observation is consistent with previous reports that SFRP1 can also be regulated through histone modification and microRNAs.^82^, ^83^ Collectively, the aforementioned studies elucidate how the reduced expression of SFRP1, SFRP2, SFRP4, and SFRP5 mediate OC chemoresistance. Nevertheless, given that the methylation profile of SFRP isoforms vary markedly across histotypes and OC cell lines,^45^ a limitation of these studies is the consideration of SFRP isoforms in isolation. To that end, investigating all SFRP isotypes simultaneously in future experiments could reveal histotype and OC stage‐associated variations, as well as any interplay and functional redundancy between isoforms.

#### 2.3.2 ZNF671

ZNF671 is a transcriptional repressor that belongs in the Kruppel‐associated box zinc finger proteins (KRAB‐ZFPs) family. Its role as a tumor suppressor has been well documented, notably in nonsmall cell lung cancer^84^ and urothelial carcinoma.^85^ Furthermore, pan‐cancer analysis using The Cancer Genome Atlas (TCGA) data set indicated that reduced ZNF671 expression is associated with promoter hypermethylation.^86^ Consistent with its role as a tumor suppressor gene, ZNF671 hypermethylation predicted poor prognosis across eight solid tumors. However, OC was not assessed in this study. To that end, Mase et al. analyzed the methylation status of 584 patients using the TCGA database: ZNF671 was the most significantly methylated gene found in early recurrence (within 12 months) OC patients.^87^ Across 78 HGSOC patients, 5/29 (17.2%) patients without ZNF671 methylation experienced early recurrence compared to 22/49 (44.9%) of patients with ZNF671 methylation. Consistent with data across other solid tumors,^86^ ZNF671 DNA methylation predicted a reduced overall survival rate.

Experiments involving siRNA‐mediated inhibition of ZNF671 across different OC cell lines have reinforced its important antitumorigenic role. In JHOS‐2 and NIH‐OVCAR3 OC cell lines, ZNF671 depletion culminated in significantly greater cell growth.^87^ In JHOS‐2 and SKOV3, siRNA‐mediated silencing culminated in enhanced cell migration and cell invasion was also greater in the latter.^87^ These findings are congruent with those from experiments performed on nonsmall cell lung cancer cells.^84^ This study also provided more insight into specific signaling pathways: GSEA analysis indicated that ZNF671 expression was associated with reduced expression of genes involved in Wnt/β‐catenin signaling. Further Western Blot analysis showed that cells overexpressing ZNF671 produced less β‐catenin as well as CyclinD1 and MMP9 (downstream Wnt/β‐catenin pathway genes). Moreover, in ZNF671 overexpressing cells, upregulation of β‐catenin reversed the reduced cell growth and migratory capacity.^84^ These results may elucidate the fact that ZNF671 is negatively associated with angiogenesis, EMT, and invasion.^88^ Indeed, upregulated Wnt/β‐catenin signaling has been widely implicated in these aforementioned protumorigenic processes.^89^, ^90^ Thus, recent developments have highlighted the potential of ZNF671 as a potential marker of disease recurrence, as well as shedding light on its involvement in signaling pathways.

### 2.4 Calcium and G protein‐coupled receptor signaling

#### 2.4.1 NCALD

NCALD is a member of the Neuronal Calcium Sensor family and is involved in calcium and G protein‐coupled receptor signaling. High levels of NCALD expression have been shown to predict poorer prognosis in acute myeloid leukemia^91^ and nonsmall cell lung carcinoma.^92^ Given that NCALD is expressed across various tissues, including the ovaries,^93^ recent attention has turned to assessing the relationship between abnormal NCALD methylation and OC chemoresistance.

In 2014, blood cell mRNA analysis from OC patients revealed that NCALD downregulation was significantly associated with reduced overall survival. Moreover, reduced NCALD expression was more prevalent in poorly differentiated tumors.^94^ Feng et al. validated these findings and also outlined that NCALD expression was significantly lower in the tumor tissue from chemoresistant OC patients.^95^ Although these studies provided transcriptomic data, neither assessed the relationship between NCALD methylation and gene expression. To that end, in a more recent study, 450 K Infinium Methylation BeadChip analysis showed that there was greater methylation at three specific CpG sites within the NCALD gene in chemoresistant compared to chemosensitive HGSOC.^96^ Notably, the sensitivity and specificity of cg27637873 methylation (candidate CpG within NCALD) to predict chemoresponsiveness were 81.8% and 78.6%, respectively. Analysis across 132 advanced HGSOC samples indicated that hypermethylation corresponded to reduced NCALD transcript and protein expression. Interestingly, NCALD hypermethylation only correlated with reduced progression‐free survival in patients with complete debulking. Accordingly, an important future avenue is to consider how different methylation markers lose their prognostic and therapeutic significance in patients with incomplete debulking.

The mechanism of NCALD involvement in OC is less well‐defined. Bioinformatic analysis elucidated that the relationship between NCALD and drug resistance may be underpinned by a plethora of biological processes including angiogenesis, cell growth, and cell cycle.^97^ Moreover, bioinformatic analysis of competing endogenous RNA (ceRNA) and mRNA interactions suggested that NCALD may regulate other genes: it can potentially behave like a ceRNA for 89 genes across over 20 different cancers.^97^ Most notably, NCALD mRNA expression correlated most strongly with CX3CL1 expression in OC tissue. Downregulation of both NCALD and CX3CL1 was associated with reduced overall survival and transcript levels were also reduced in chemoresistant HeyA8 and SKOV3 cell lines.^97^ Although CX3CL1 has been heavily implicated in both protumorigenic and antitumorigenic processes, these findings would suggest that CX3CL1 has a predominantly anti‐tumorigenic function in OC. For instance, it may promote antitumour immune cell (e.g., NK cell, CD8+ T cells) infiltration into the tumor microenvironment.^97^, ^98^ Nevertheless, the aforementioned studies lack a CX3CL1 knockout sample, thus it cannot yet be concluded to what extent aberrant NCALD and CX3CL1 expressions are “driver‐like” events for chemoresistance. Regardless, the NCALD methylation status appears to be strongly associated with OC prognosis and warrants further investigation.

### 2.5 DNMTi—Evidence from clinical trials

**Figure 3:**
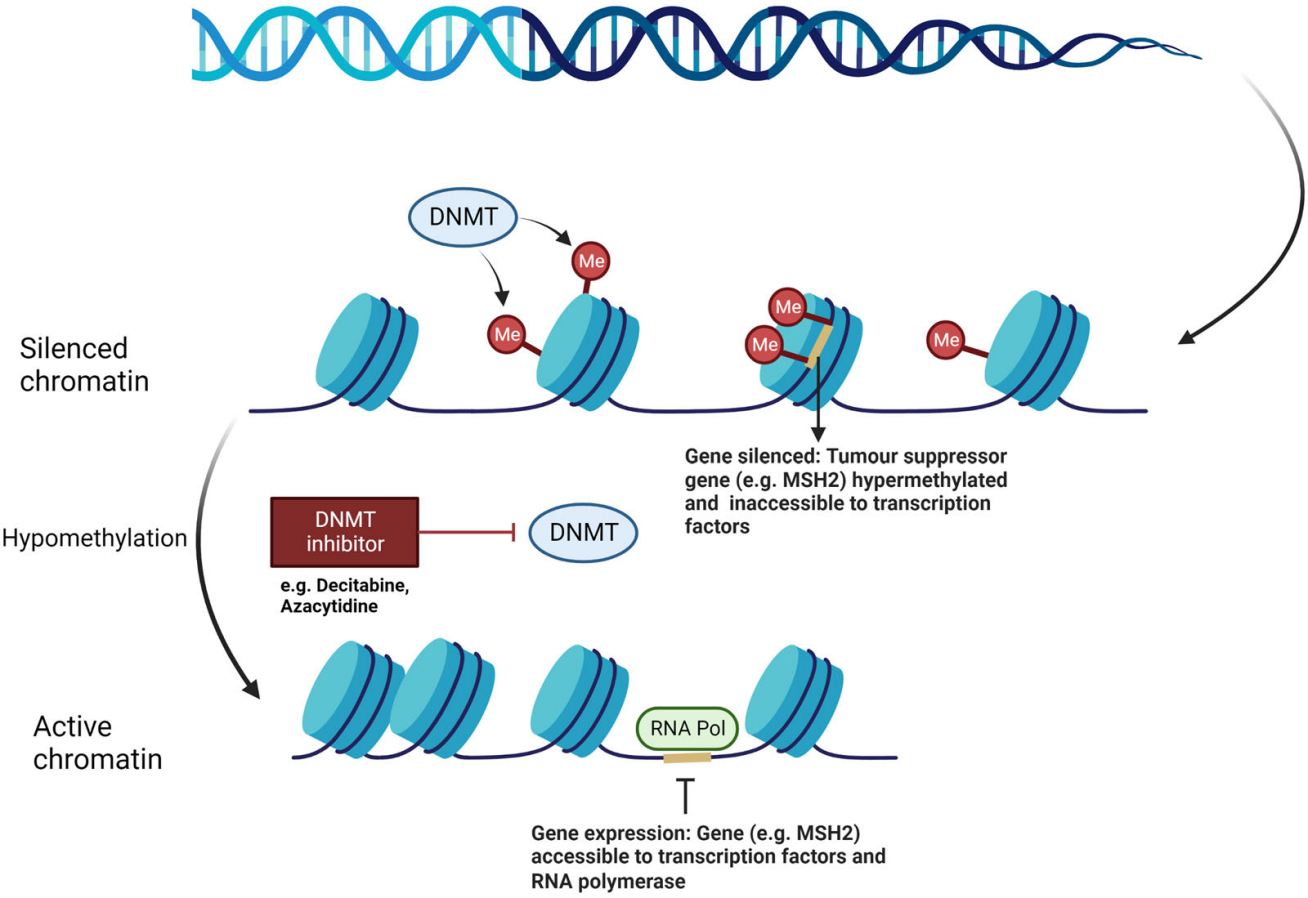
Schematic showing the role of methylation on chromatin structure (acetylation events are not shown here). DNA methyltransferase inhibitors (DNMTis) inhibit DNA methyltransferases (DNMTs). Subsequent hypomethylation promotes a more open chromatin configuration that is accessible for transcription machinery. Thus, in theory, DNMTi therapy could upregulate the expression of previously hypermethylated and underexpressed, tumor suppressor genes. [Created with BioRender.com].

Given that DNA hypermethylation, unlike genetic mutations, can be readily reversed, there has been significant therapeutic interest surrounding DNMTi (Figure 3). Azacytidine and decitabine were the first Food and Drug Administration‐approved DNMTi, having been authorized for treating myelodysplastic syndrome in 2004 and 2006, respectively.^99^ These DNMTi are cytidine analogs that interact irreversibly with DNMTs and promote their proteasomal degradation.^100^ The emergence of preclinical data showing that DNMTi demethylates critical genes including SFRPs,^44^ MLH1,^31^ and RASSF1A,^101^ while sensitizing chemoresistant OC cells and xenograft models has inspired several clinical trials (Table 2).

**Table 2:** Clinical trials involving combined DNMTi and platinum treatment in chemoresistant OC patients.

Clinical trial type and year	Demethylating agent	Chemotherapeutic agent	Notable genes that were hypomethylated following treatment	Clinical outcome in platinum‐resistant ovarian cancer patients	References		
Phase I (2010)	Decitabine	Carboplatin	HOXA10, BRCA1	1/19 CR, 3/19 SD (>6 months)	[102]		
Phase I (2011)	Azacytidine	Carboplatin	N/A	1/18 CR, 3/18 PR, 10/18 SD, ORR 22%	[103]		
Phase II (2012)	Decitabine	Carboplatin	MLH1, HOX10, HOX11, RASSF1A	1/17 CR, 5/17 PR, 6/17 SD, ORR 35%	[104]		
Phase II (2017)	Decitabine	Carboplatin + paclitaxel	N/A	1/40 CR, 8/40 PR, 19/40 SD, ORR 23%	[105]		
Phase I (2018)	Guadecitabine	Carboplatin	FOK2, RASSF1A, FZD1	3/20 CR, 6/20 SD, ORR 15%	[106]		
Phase II (2020)	Guadecitabine	Carboplatin	MAGE‐A2, MAGE‐A3	8/51 CR or PR, ORR 16%	[107]		

Abbreviations: CR, complete response; DNMTi, DNA methyltransferase inhibitors; PR, partial response; ORR, objective response rate; SD, stable disease.

After an initial phase I trial showed that low‐dose decitabine was well‐tolerated and induced global hypomethylation in chemoresistant OC patients,^102^ a follow‐up phase II trial showed that decitabine sensitized patients to carboplatin. 6/17 patients exhibited either a complete or partial response,^104^ a markedly higher objective response rate (ORR) than is expected with sole carboplatin retreatment (<10%).^108^, ^109^ Significantly, both MLH1 and RASSF1A decitabine‐induced demethylation positively correlated with progression‐free survival,^104^ reinforcing the significance of their methylation profiles in chemoresistance. Nevertheless, a phase II trial involving single‐dose decitabine preceding carboplatin treatment was prematurely terminated owing to a lack of efficacy and frequent hypersensitivity reactions and neutropenia.^110^ This finding is elucidated by the fact that, unlike with prolonged low‐dose DNMTi treatment, a single high‐dose administration of DNMTi is unlikely to mediate prolonged demethylation but more likely to elicit dose‐limiting toxicities.^111^

The recent emergence of a second‐generation DNMTi, guadecitabine, could enhance the feasibility of combined DNMTi and platinum therapy. Guadecitabine is a dinucleotide of decitabine and deoxyguanosine that possess a longer half‐life than decitabine/azacytidine,^112^ owing to its resistance to cytidine deaminase‐mediated degradation. In an initial phase I trial, guadecitabine preceded by carboplatin was tolerated and elicited an ORR of 15% among heavily pretreated patients.^106^ The dose and schedule of combined guadecitabine and carboplatin treatment employed has since been validated by similar phase I studies based on germ cell cancer^113^ and urothelial carcinoma.^112^

Subsequently, a randomized phase II trial based on 100 platinum‐resistant OC patients receiving either guadecitabine and carboplatin (G + C) or a nonplatinum treatment of choice (TC) ensued.^107^ Although the study did not reach the intended primary endpoint (median progression‐free survival [PFS]), the 6‐month PFS rate was significantly greater in the G + C cohort (37% compared to 11%). Notably, this trial lacked a sole carboplatin treatment control group, thus it is difficult to ascertain to what extent guadecitabine was responsible for improved clinical outcomes.

### 2.6 Future direction of DNMTi and methylated‐based biomarkers

Although combined DNMTi and chemotherapy has shown promise, concerns surrounding the nonspecific nature of global hypomethylation and dose‐limiting toxicities remain. Given that DNMTi therapy is not feasible for patients whose chemoresistance is mediated by hypomethylation or methylation‐independent events, a greater appreciation of chemoresistance‐associated methylation profiles is a requisite step preceding the clinical feasibility of DNMTi. Indeed, the limited efficacy observed with guadecitabine and platinum therapies is likely attributed to the fact that patients cannot yet be selected for DNMTi therapy based on predictive methylation markers. Ideally, in the future, the prediction of chemoresistance and subsequent suitability for DNMTi therapy could be informed by sampling OC patient's plasma cell‐free tumor DNA (cfDNA) for specific methylation‐based biomarkers.

The use of liquid biopsies to assess cell‐free DNA has come to the fore in recent years: it represents a noninvasive, safe, and convenient technique that could transform OC diagnosis and management. Crucially, cfDNA methylation changes are present during the early stages of carcinogenesis and are highly concordant with the methylome of the primary tumor.^40^, ^114^ Thus, analysis of cfDNA methylation markers could facilitate early OC diagnosis and indicate chemoresistance. Indeed, this approach has already shown significant potential in the context of diagnosing OC and differentiating benign and malignant tumors.^115^, ^116^ More recently, cfDNA analysis has been used to demonstrate that the reversion of BRCA1/2 mutations in HGSOC patients was associated with platinum and PARP inhibitor resistance.^117^ Although several studies have analyzed the relationship between cfDNA methylation markers and OC chemosensitivity, these have predominantly focused on a single gene, for example, MCJ,^28^ SFRP5.^43^ Altogether, these developments indicate the diagnostic, therapeutic, and monitoring potential of studying liquid biopsies in OC patients. Nevertheless, to utilize cfDNA methylome analysis to assess OC chemosensitivity, a greater appreciation of chemoresistance‐associated methylation biomarkers is required.

To that end, a large‐scale prospective genome‐wide DNA methylation profile study, expanding upon one recently performed in advanced HGSOC,^96^ is warranted. Importantly, this study should include different OC histotypes and integrate both transcriptomic and methylomic analysis. Paired tumor samples from responders and non‐responders should be studied before and after chemotherapy. In addition to the genes discussed, this approach would highlight more differentially methylated candidate genes, whose roles in chemoresistance could be elucidated via Ingenuity Pathway Analysis. Subsequently, a panel of gene methylation profiles could be refined; combinations of specific methylation signatures could be derived, which predict chemoresponsiveness with high specificity and sensitivity. Further investigations can then reveal whether the target methylation markers found in the primary tumor are mirrored in cfDNA samples. Altogether these developments could facilitate the use of noninvasive serum sampling to identify OC patients susceptible to DNA hypermethylation‐associated relapse/chemoresistance who would benefit from DNMTi therapy. This personalized approach could optimize combined DNMTi and chemotherapy approaches and minimize the prevalence of nonresponders and dose‐limiting toxicities.

## 3 CONCLUSION

Here, we have discussed how epimutations across key protumorigenic processes, including drug export, apoptosis, and Wnt/β‐catenin signaling, are linked with OC chemoresistance. Notably, methylation events that upregulate the expression of ABC transporters (e.g., ABCB1 and ABCG2) confer resistance through promoting chemotherapeutic drug efflux. Moreover, hypermethylation events that downregulate the expression of proapoptotic (e.g., RASSF1A) and/or DNA repair genes (e.g., MLH1, MSH2) are associated with chemoresistance in clinical OC samples. Aberrant methylation can also promote pro‐oncogenic pathway signaling (e.g., Wnt/β‐Catenin) through altering the expression of regulators of such processes (e.g., SFRPs, ZNF671). Finally, propelled by bioinformatic advances, some signaling proteins (e.g., NCALD), which have previously been associated with other cancers, are increasingly being seen in a new light; such genes could represent novel biomarkers, however, their specific functional/metabolic roles in OC remain largely unknown.

Although a myriad of clinically and statistically significant data associate these biomarkers with OC chemoresistance, there are still considerable gaps in our knowledge relating to the epimutations discussed (summarized in Table 3). Examples of largely unexplored domains include how biomarker expression varies prechemotherapy versus postchemotherapy (i.e., intrinsic vs. acquired resistance), disparities across OC histotypes, and the significance of the specific site of methylation within the promoter region(s). Given these limitations, it is unlikely that the maximal potential of these prognostic biomarkers will be realized without further work. Ideally, once these key domains have been addressed across the candidate biomarkers, further experiments that employ sensitive, quantitative methylation sequencing techniques and liquid biopsy technology can eventually facilitate OC patient stratification based on factors including tumor subtype and stage, whether chemotherapy and/or debulking have been initiated, and epigenetic profile. These advancements would likely lead to a greater understanding of OC patients' suitability for DNMTi therapy, ultimately improving the efficacy of DNMTis and reducing unnecessary side effects.

**Table 3:** Summary of selected genes discussed.

Gene and typical methylation profile in chemoresistance	Gene function	Current unanswered questions and/or important areas of consideration	Proposed future direction
ABCB1 (hypomethylation/hypermethylation)	Drug export and sustains “side population” cells	Studies into ABCB1 methylation status in OC patients are lacking	Future studies on OC patients should specify the ABCB1 promoter and integrate both methylomic and transcriptomic analysis
		Downstream ABCB1 promoter hypermethylation upregulates ABCB1 expression	
DNAJC15 (hypermethylation)	Regulates ABCB1 expression	Differential methylation patterns across histotypes	Greater histotype‐specific methylome analysis is required
MLH1 and MSH2 (hypermethylation)	DNA mismatch repair and triggers apoptosis in response to DNA adducts	Unlike with MLH1, it is less well‐established whether MSH2 methylation is associated with intrinsic or acquired resistance	MSH2 methylation status should be assessed in OC patients' both before and after chemotherapy
RASSF1A (hypermethylation)	Promotes microtubule stabilization and apoptosis	Marked variation of RASSF1A hypermethylation prevalence across OC patients	Greater use of sensitive, quantitative sequencing approaches, for example, pyrosequencing
SFRPs (hypermethylation)	Wnt/beta‐catenin signaling promotes twist/Akt‐2 signaling	The interplay between SFRP isoforms and the variation in their methylation profiles across different histotypes and tumor grades is largely unknown	Future OC patient‐based studies on SFRPs should investigate the methylome of all 5 SFRP isoforms
NCALD (hypermethylation)	Member of the neuronal calcium sensor family, involved in calcium and G protein‐coupled receptor signaling	Relationship between hypermethylation and prognosis/chemosensitivity is predominantly evident in patients with complete debulking	The precise functional role of NCALD in ovarian cancer chemoresistance remains elusive and requires investigating
ZNF671 (hypermethylation)	Transcriptional repressor belonging to the Kruppel‐associated box zinc finger proteins (KRAB‐ZFPs) family	The precise signaling interactions and pathways that ZNF671 is implicated in, specifically in the context of OC	Is there a synergistic relationship between the hypermethylation of ZNF671 and other Wnt/β‐catenin genes in OC chemoresistance?

Abbreviation: OC, ovarian cancer.

## AUTHOR CONTRIBUTIONS

**Kaiyang Song**: Investigation; visualization; writing—original draft. **Mara Artibani**: Supervision; writing—review and editing.

## CONFLICT OF INTEREST STATEMENT

The authors declare no conflict of interest.

## ETHICS STATEMENT

No animals or clinical samples were used in this study.

## TRANSPARENCY STATEMENT

The lead author Mara Artibani affirms that this manuscript is an honest, accurate, and transparent account of the study being reported; that no important aspects of the study have been omitted; and that any discrepancies from the study as planned (and, if relevant, registered) have been explained.

## Supporting information

Supplementary information.

## Data Availability

Mara Artibani and Kaiyang Song had full access to all the data in this study and takes complete responsibility for the integrity of the data and the accuracy of the data analysis.
