# Supplementary material for: The role of DNA methylation in ovarian cancer chemoresistance: A narrative review
Source: Health Sci Rep. 2023 Apr 27;6(5):e1235. doi: 10.1002/hsr2.1235 (PMC10140645; doi:10.1002/hsr2.1235)
Supplement: Supplementary file 1 — Supplementary information. [file HSR2-6-e1235-s001.docx]

Figure S1: Flow chart depicting the literature selection process used

Studies included in review

Excluded studies based on: relevance, study design, full text accessibility

Duplicates removed n

Excluded Studies based on: study design, relevance and language (i.e. not in English)

Full text articles assessed for eligibility

Google Scholar

Title and abstract screening

Studies identified through database searching

PubMed
